# Supplementary material for: Classical harmonic three-body system: an experimental electronic realization
Source: Sci Rep. 2022 Aug 3;12:13346. doi: 10.1038/s41598-022-17541-0 (PMC9349263; doi:10.1038/s41598-022-17541-0)
Supplement: Supplementary file 1 — Supplementary Information. [file 41598_2022_17541_MOESM1_ESM.pdf]

Supplementary Material
Classical harmonic three-body system: An experimental electronic realization

A. M. Escobar-Ruiz,[1] M. A. Quiroz-Juarez,[2] J. L. Del Rio-Correa,[1] and N. Aquino[1]

[1] *Departamento de Física, Universidad Autónoma Metropolitana Unidad Iztapalapa, San Rafael Atlixco 186, 09340 Cd. Mx., México*

[2] *Centro de Física Aplicada y Tecnología Avanzada, Universidad Nacional Autónoma de México, Boulevard Juriquilla 3001, Juriquilla, 76230 Querétaro, México*

(Dated: July 22, 2022)

In this supplementary material, we show the scaling mass relation in the classical three-body harmonic system.

I. SCALING MASS RELATION

One can also use the variables $(L_1,\, L_2,\, \sigma)$

$$
\begin{aligned}
r_{12}^2 &= (\,L_2^2 \,-\, L_1^2\,)\sin\sigma\,\cos\sigma \\
r_{13}^2 &= L_2^2\,\sin^2\sigma \,+\, L_1^2\,\cos^2\sigma \\
r_{23}^2 &= L_2^2\,\cos^2\sigma \,+\, L_1^2\,\sin^2\sigma\ ,
\end{aligned}
\tag{1}
$$

(see Ref.[1] and references therein) which are useful in the study of the general three-body problem. In these variables, the potential (2) of the main manuscript takes the form

$$
\begin{aligned}
V \;=\; 2\,\omega^2\Bigg[\nu_{12}\left(\sqrt{(\,L_2^2\,-\,L_1^2\,)\sin\sigma\,\cos\sigma}\,-\,R_{12}\right)^2 \\
+\;\nu_{13}\left(\sqrt{L_2^2\,\sin^2\sigma\,+\,L_1^2\,\cos^2\sigma}\,-\,R_{13}\right)^2 + \\
\nu_{23}\left(\sqrt{L_2^2\,\cos^2\sigma\,+\,L_1^2\,\sin^2\sigma}\,-\,R_{23}\right)^2\Bigg]\ .
\end{aligned}
\tag{2}
$$

Accordingly, the reduced Hamiltonian (1) of the main manuscript becomes

$$
\mathcal{H} \;=\; \frac{1}{2\,\mathcal{M}}\left(P_{L_1}^2 \,+\, P_{L_2}^2 \,+\, \frac{L_2^2\,+\,L_1^2}{(L_2^2\,-\,L_1^2)^2}\,P_\sigma^2\right) + V\ , \tag{3}
$$

here $\mathcal{M} \;\equiv\; \sqrt{\frac{m_1\,m_2\,m_3}{m_1+m_2+m_3}}$. Eventually, the following scaling relation emerges

$$
\mathcal{H}[\,\mathcal{M},\,\omega\,] \;=\; \frac{\tilde{\mathcal{M}}}{\mathcal{M}}\,\mathcal{H}[\,\tilde{\mathcal{M}},\,\omega\,\mathcal{M}/\tilde{\mathcal{M}}\,]\ , \tag{4}
$$

where $\tilde{\mathcal{M}} \;\equiv\; \sqrt{\frac{\tilde{m}_1\,\tilde{m}_2\,\tilde{m}_3}{\tilde{m}_1+\tilde{m}_2+\tilde{m}_3}}$. It allows us to connect two systems with different masses. Unlike the ρ-representation, the Hamiltonian $\mathcal{H}$ is not a polynomial function in the variables $(L_1,\, L_2,\, \sigma)$.

[1] E. Piña and L. Jiménez-Lara, *Perturbing the Lagrange Solution to the General Three Body Problem* (Springer, Boston, MA, 2004) pp. 123–136.
